# Endothelin‐Converting Enzyme‐Like 1 Regulated by LIF Contributes to Chronic Constriction Injury‐Induced Neuropathic Pain in Mice

**DOI:** 10.1111/cns.70578

**Published:** 2025-09-04

**Authors:** Feng Gao, Yuchen Pan, Yong Huang, Chen Gu, Xiaowei Song, Cunjin Wang

**Affiliations:** ^1^ Department of Orthopedics Jiangsu Provincial Corps Hospital, Chinese People's Armed Police Force Yangzhou China; ^2^ Department of Neurology Jiangsu Provincial Corps Hospital, Chinese People's Armed Police Force Yangzhou China; ^3^ Department of Neurosurgery Jiangsu Provincial Corps Hospital, Chinese People's Armed Police Force Yangzhou China; ^4^ Clinical Medical College Yangzhou University Yangzhou China; ^5^ Department of Anesthesiology Northern Jiangsu People's Hospital Yangzhou China; ^6^ Northern Jiangsu People's Hospital Affiliated to Yangzhou University Yangzhou China; ^7^ The Yangzhou Clinical Medical College Xuzhou Medical University Yangzhou China

**Keywords:** DRG, ECEL1, LIF, neuropathic pain, sympathetic sprouting

## Abstract

**Aims:**

This study is to investigate the role of Endothelin‐converting enzyme‐like 1 (ECEL1) in neuropathic pain (NP).

**Methods:**

The expression of ECEL1 was modulated by injecting adeno‐associated virus 5 (AAV5) carrying Ecel1 shRNA or full‐length Ecel1 into the dorsal root ganglion (DRG) of mice with a chronic constriction injury (CCI) model. Then, various nociceptive responses were evaluated. Additionally, leukemia inhibitory factor (LIF) was intrathecally injected, or its function was blocked, to observe the changes in ECEL1 expression.

**Results:**

Our findings demonstrate that downregulating ECEL1 expression alleviates CCI‐induced pain and reduces the hyperexcitability of injured DRG neurons, which is achieved by inhibiting sympathetic sprouting in the DRG. Conversely, overexpressing ECEL1 in DRG neurons leads to pain hypersensitivity. Additionally, we observed that LIF upregulated ECEL1 expression, while blocking LIF reduced ECEL1 expression and mitigated CCI‐induced nociception in mice.

**Conclusion:**

ECEL1 promotes hyperalgesia following CCI and is regulated by LIF, suggesting it could be a new target for NP treatment.

## Graphical abstract

After chronic constriction injury (CCI), leukemia inhibitory factor (LIF) is transported from the distal injury site to dorsal root ganglion (DRG) neurons and promotes the expression of ECEL1, thereby promoting sympathetic sprouting and causing neuropathic pain.

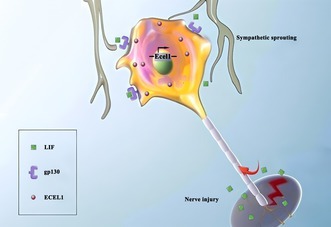

## 1 Introduction

Neuropathic pain (NP), a type of persistent pain directly caused by damage or disease affecting the somatosensory system [1], is characterized by spontaneous pain, hyperalgesia, tactile allodynia, and paresthesia. The prevalence of chronic NP in the general population has been reported to be as high as 6.9%–10% [2]; yet effective treatment options remain limited [3]. NP significantly impacts the physical, psychological, and social well‐being of patients. Prolonged chronic pain often leads to anxiety, depression, and suicidal tendencies among patients, posing a significant threat to their survival [4]. Although there is some understanding of the complex causes of NP and the related mechanisms, which involve various neurotransmitter systems, receptors, ion channels, and cell types, the management of NP remains challenging [1]. Therefore, a comprehensive investigation of the pathogenesis of NP, especially its molecular mechanism, is crucial for identifying novel therapeutic targets and developing more effective treatment strategies.

The dorsal root ganglion (DRG) serves as a crucial relay station for the transmission of nociceptive signals from the periphery to the central nervous system [5]. When peripheral nociceptive signals reach the DRG, they trigger numerous changes in the expression of pro‐ and antinociceptive genes. Endothelin‐converting enzyme‐like 1 (Ecel1), a metallopeptidase first identified in 1999 [6], is specifically expressed in neurons of both the central and peripheral nervous systems. Since its discovery, ECEL1 has been the subject of growing research interest due to its roles in nerve damage responses and neural development. Its expression is induced in response to nerve damage, such as damage induced by cranial and spinal nerve transection, ischemia, corpus callosum transection, and colchicine treatment [7]. In the context of these conditions, ECEL1 mRNA expression significantly increases in injured neurons. Thus, it is also referred to as damage‐induced neuronal endopeptidase (DINE) [8, 9].

Previous research has demonstrated that Ecel1 plays a significant role in neural development. For example, mice with an introduced pathogenic mutation in Ecel1 display defects in the axonal arborization of motor nerves in the extremities, along with dysplastic neuromuscular junctions, leading to distal arthrogryposis [10]. Additionally, mutations in the ECEL1 gene within human populations lead to abnormal neurodevelopment and a significant reduction in nerve terminal dendrites, leading to motor developmental delay and joint contractures [11]. On the other hand, Ecel1 gene expression is coregulated by axon regeneration‐related transcription factors, including ATF3, c‐Jun, and STAT3. It promotes nerve regeneration in injured neurons and exerts neuroprotective effects [9, 12, 13]. Taken together, these findings indicate that ECEL1 plays important roles in both the development of neural tissue and the regeneration of injured nerves.

While ECEL's functions in development and regeneration are well‐established, its role in neuropathic pain remains unclear. Our initial exploration, based on RNA‐Seq data analysis of DRG tissue samples from chronic constriction injury (CCI) model mice, revealed a notable increase in ECEL1 expression in the DRG. Considering the critical role of the DRG in nociceptive signal transmission and the known functions of ECEL1 in nerve‐related processes, we hypothesized that ECEL1 may play an important role in the pathological process of NP development. This study aims to experimentally test this hypothesis and elucidate the underlying mechanisms, potentially paving the way for new therapeutic approaches for NP.

## 2 Materials and Methods

### 2.1 Animals

We obtained C57BL/6 mice weighing 25–30 g (8–12 weeks) from the Experimental Animal Center of Yangzhou University. Prior to the experiment, all the experimental animals were allowed a 2‐week acclimation period. The animal experiments conducted in this study adhered to the National Institutes of Health Guidelines on the Use of Laboratory Animals and were approved by the relevant institutional committee on animal care.

### 2.2 NP Model

The CCI surgery was performed following Bennett and Xie's protocol [14]. Mice were anesthetized with 40 mg/kg pentobarbital sodium via intraperitoneal injection. The skin in the middle of the left thigh was cut; the muscle between the superficial gluteal muscle and the biceps femoris was separated using sharp forceps. A nerve peeler was used to pick out the sciatic nerve, which was then loosely ligated with two6‐0 silk sutures 1 mm from its trifurcation. The incision was closed in layers with absorbable sutures.

### 2.3 Behavioral Analysis

Mechanical pain was evaluated by Von Frey hairs to determine the mechanical withdrawal threshold (MWT) [15]. Mice were placed in a Plexiglas cage with a wire mesh floor and allowed to acclimate for 30 min before testing. Seven calibrated Von Frey fiber filaments (0.04, 0.07, 0.16, 0.4, 0.6, 1.0, and 1.4 g) were used for the test, starting from the lowest weight and stimulating the center of the left hind paw's plantar surface vertically with each filament for 3 s when it was slightly bent. Each filament was measured five times at 5‐min intervals. The MWT was defined as the lowest filament force in grams that produced at least three withdrawal responses in five tests.

Thermal hyperalgesia was assessed using an IITC Plantar Analgesia Meter to measure the thermal withdrawal latency (TWL) [16]. The mice were placed in a Plexiglas cage with a wire mesh floor and were allowed to habituate for 30 min before testing. The test was carried out by irradiating the central part of the plantar surface of the hind paw using the thermal radiation light source under the transparent glass. The stimulus was shut off when the hind paw moved, or after 20 s to prevent tissue damage. The time from the onset of radiant heat to the endpoint was the TWL. Thermal stimuli were delivered three times to each hind paw at 5‐min intervals.

Cold hyperalgesia measurements were conducted via a previously established method [16]. Mice were placed in individual Plexiglas chambers positioned on a 2.5‐mm‐thick elevated glass platform. Cold stimulation to the plantar surface of the hind paw was applied from beneath the platform by placing dry ice in gentle contact with the glass, ensuring a smooth ice surface to avoid uneven cooling. Withdrawal latency was recorded using a stopwatch, defined as the time until the paw is lifted or moved away from the cold surface. A 20‐s cutoff was implemented to prevent tissue injury, and each hind paw was subjected to three cold stimuli at 5‐min intervals.

### 2.4 Rotarod and CatWalk Automated Gait Analysis

Place the mice on a rotarod (Ugo Basile) that can accelerate rotation. The rotarod is set to accelerate from 5 to 40 rpm for a duration of 300 s. After 3 days of training, take the average of the time the mice remain on the rotarod in the three consecutive trials.

The CatWalk XT system (Noldus Information Technology, Wageningen, The Netherlands) was used to evaluate gait parameters and footfalls in rodents. It is validated in various pain models and is reliable in measuring nociceptive behavior [17]. Mice traversed a darkened glass‐plate tunnel (100 × 10 cm) illuminated from below by fluorescent light. Footprints were captured at 200 fps by a high‐speed camera positioned beneath the plate, with data processed using proprietary software. Inclusion criteria for valid runs were: (i) transit time through the imaging zone of 0.5–5 s, and (ii) ≤ 60% variability in run velocity. Analyzed parameters included: (1) print area (contact surface area between hind paw and glass, mm^2^), and (2) single stance duration (time during which a hind paw maintains continuous contact with the glass, ms), both of which serve as indices of nociceptive behavior.

### 2.5 DRG and Intraspinal Microinjection

DRG microinjection was carried out following established procedures [18]. After the mice were anesthetized with sodium pentobarbital, a midline incision was made in the lower lumbar region to expose and remove the lumbar articular process. The exposed DRG was then injected with adeno‐associated virus 5 (AAV5) containing Ecel1 short hairpin RNA (shRNA) (AAV5‐Ecel1‐shRNA‐GFP) (3.4E + 12 v.g./mL), gp130 short hairpin RNA (shRNA) (AAV5‐gp130‐shRNA‐GFP) (3.2E + 12 v.g./mL), a green fluorescent protein expression marker (AAV5‐scrambled‐shRNA‐GFP) (3.8E + 12 v.g./mL), AAV5 expressing full‐length Ecel1 (AAV5‐Ecel1‐overexpression‐GFP) (3.5E + 12 v.g./mL), or AAV5‐GFP (3.4E + 12 v.g./mL) as a control. A 0.5 μL volume of the viral solution was injected through a glass micropipette connected to a microsyringe filled with mineral oil; after injection, the pipette was held in place for 10 min. The shRNA targeting the sequence of mouse Ecel1 (Gene Bank Accession NM_001277925.2) was designed as follows: 5′‐GAGTTCCAGGAAGTCAAGTATCTCGAGATACTTGACTTCCTGGAACTC‐3′. The shRNA targeting the sequence of mouse gp130 (Gene Bank Accession NM_010560.3) was designed as follows: 5′‐CAAAGTGTGTCTGAGTTTATACTCGAGTATAAACTCAGACACACTTTG‐3′. A scrambled sequence was also designed as a negative control (Scram): 5′‐ATTGGATGCGATCTGCGCCTGTATACAGATTCGAGATCTAACACTACG‐3′. The AAV vectors were designed and generated by Shanghai Taitool Bioscience.

Intrathecal catheterization was performed as described before [19]. Under isoflurane anesthesia, the atlanto‐occipital membrane was exposed by separating the superficial neck muscles. A PE‐10 catheter was inserted into the subarachnoid space at the C1–C2 level, with the tip positioned near the lumbar enlargement. The catheter was secured to the skull with dental cement. From day 1 post‐CCI, mice received daily intrathecal injections of LIF (3 μg/kg/day) or vehicle (ACSF) via the catheter. Mouse recovery included observing for irritation in the first 24 h, with free access to food and water on the cage floor.

### 2.6 RNA Isolation and qPCR Analysis

The DRGs of the experimental mice were lysed with TRIzol reagent, and total RNA was extracted. Real‐time quantitative PCR (qPCR) with SYBR Green Real‐time PCR Master Mix was used to detect mRNA levels. Gene expression was normalized to *Gapdh* via the ΔΔCT method. The PCRs were: 95°C for 3 min; 45 cycles at 95°C for 10 s, 65°C for 30 s, 72°C for 10 s; and final extension at 72°C for 10 min. The primers used for ECEL1 were as follows: F: CGGAGGAGGAGGAGGTGGTG; R: GAGGACAGGTGCTCACTCAGAAC. The primers used for LIF were as follows: F: GGCAACGGGACAGAGAAGACC; R: GCTTGACCTGGAGGCTCACG. The primers used for GAPDH were as follows: F: CCATCAACGACCCCTTCATT; R: ATTCTCAGCCTTGACTGTGC.

### 2.7 RNA Sequencing and Bioinformatics Analysis

RNA sequencing and bioinformatics analysis involved extracting total RNA with an miRNeasy Mini Kit. The quantified total RNA was purified before preparing RNA‐Seq libraries per the Illumina TruSeq protocol. Gene expression was in FPKM units. A gene was expressed if FPKM value > 1; differential expression was needed > 2‐fold change and *p* < 0.05.

GO functional analysis and KEGG pathway enrichment analysis of the DEGs were conducted using online tools such as the Database for Annotation, Visualization and Integrated Discovery (DAVID) and the KEGG Orthology‐Based Annotation System (KOBAS) available at http://www.geneontology.org and http://www.genome.jp/kegg, respectively. Hierarchical cluster analysis of enriched genes was performed with Cluster 3.0 software.

### 2.8 Immunohistochemical Staining

For TH staining, mice were anesthetized and perfused with 4% PFA. Tissues were frozen and sectioned for histological analysis. Three to five slices were randomly selected. The coronal sections were permeabilized, blocked, and incubated overnight at 4°C with primary rabbit TH antibody (Abcam, 1:100). Next, the sections were washed in PBS and incubated with secondary antibody (Jackson ImmunoResearch, 1:200) at room temperature for 2 h.

The immunoreactivity of TH fibers in each DRG was quantified to estimate the number of DRG neurons enveloped by fibers with tyrosine hydroxylase immunoreactivity (TH‐IR). Neurons surrounded by TH‐IR baskets that extended beyond 50% of their perimeter were counted [20]. The total number of neurons per section was also determined. The samples were subsequently examined via confocal microscopy (FluoView FV1000; Olympus, Tokyo, Japan), and images were captured. For each sample, three spinal cord sections were selected, with six mice tested in each experimental group.

### 2.9 Western Blot Analysis

Western blot analysis was performed following previously described methods [21]. Primary antibodies, including rabbit anti‐ECEL1 (ab228490, 1:500; Abcam) and rabbit anti‐LIF (ab113262; Abcam; diluted to 1:1000), were used. The membranes were incubated with horseradish peroxidase (HRP)‐conjugated secondary antibodies (Kangcheng Biotechnology, Guangzhou, China) at a dilution of 1:5000 and horseradish peroxidase‐conjugated anti‐GAPDH (Kangcheng Biotechnology) at a dilution of 1:10000 for 2 hours. The protein bands were analyzed and quantified using Bio‐Rad Image Lab analysis software.

### 2.10 Whole‐Cell Patch Clamp Recordings

Four weeks after virus injection into the DRG, we isolated the DRG and prepared fresh dissociated cultures. The cells were plated on poly‐D‐lysine‐coated glass coverslips immersed in neural basal medium. Then, they were incubated in a cell culture incubator at 37°C with 5% CO_2_ for 24 h before the experiment. Whole‐cell patch clamp recordings were performed at room temperature using an Axonpatch 200B amplifier and a Digidata 1440A digitizer (Axon Instruments, USA) to measure action potentials and resting membrane potentials. The recording chamber (300 μL) was continuously perfused at a flow rate of 1–2 mL/min. The series resistance was compensated (> 80%), and leakage subtraction was performed. Only green fluorescent protein‐positive cells were selected for study. The pipette mixture contained the following (in mM): 126 K‐gluconate, 10 NaCl, 1 MgCl2, 10 EGTA, 2 Na‐ATP, and 0.1 Mg‐GTP, adjusted to pH 7.3 with KOH. The external solution contained the following components (in mM): 140 NaCl, 5 KCl, 2 CaCl2, 1 MgCl2, 10 N‐2‐hydroxyethyl piperazine‐N‐2‐ethanesulfonic acid, and 10 glucose, adjusted to pH 7.4 with NaOH. In the current clamp experiment, action potentials were induced by current injection steps. The resting membrane potential was measured without current injection. In the current clamp experiment, we measured the resting membrane potential when there was no current and induced action potentials via current injection steps.

### 2.11 Statistical Analysis

The data are presented as the means ± SEMs, and differences were considered significant at *p* < 0.05. Two‐tailed Student's t tests were used for statistical comparisons of two groups. One‐way and two‐way analysis of variance (ANOVA) with Tukey's post hoc test were employed to evaluate the changes in mRNA and protein expression, as well as the outcomes of the behavioral and electrophysiology studies. We used the Kolmogorov–Smirnov test to assess the normality of our data. For data that did not exhibit a normal/Gaussian distribution, we employed nonparametric equivalent analyses. All the statistical analyses were conducted using GraphPad Prism 8.3.0 software.

## 3 Results

### 3.1 Establishment of the CCI Model and Evaluation of the Gene Expression Profile of the DRG

**FIGURE 1:**
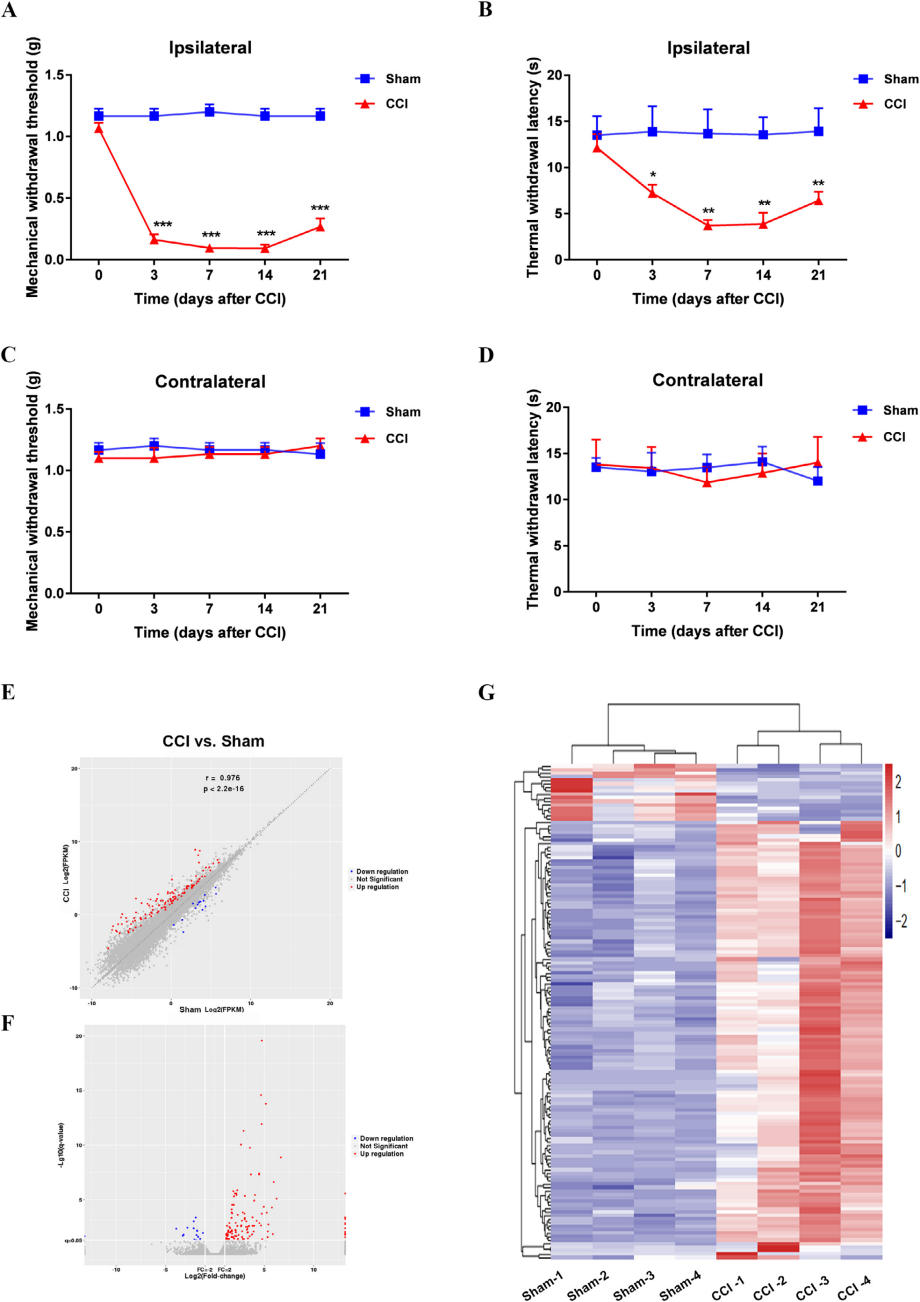
Analysis of DEGs in the DRG in the context of neuropathic pain induced by CCI. (A–D) Assessment of paw withdrawal responses to mechanical stimuli (A, C) and thermal stimuli (B, D) on the ipsilateral and contralateral sides of mice subjected to CCI compared with those in the sham group. *n* = 6 per group. (E) Upregulated and downregulated genes in the ipsilateral L4 and L5 DRGs in the CCI group compared with the sham group. (F) Volcano plot showing the upregulated and downregulated genes in the ipsilateral L4 and L5 DRGs in the CCI group compared with the sham group. The red dots represent genes whose expression was significantly upregulated, the blue dots represent genes whose expression was significantly downregulated, and the gray dots represent genes whose expression was not significantly different. (G) Heatmap showing the hierarchical clustering of DEGs in the CCI group compared with the sham group. The results are expressed as the mean ± SEM; *p < 0.05, **p < 0.01, ***p < 0.001 versus the sham group. Two‐way ANOVA with Tukey's post hoc test was used in A–D.

CCI surgery was performed to establish a mouse model of NP, as previously described [14]. CCI mice exhibited increased mechanical and thermal hyperalgesia in the ipsilateral hind paw from days 3 to 21 compared with sham controls (Figure 1A,B). The contralateral hind paw showed no significant hyperalgesia in CCI mice (Figure 1C,D). To explore the genes that might be involved in the induction of the CCI‐induced NP model, we harvested ipsilateral L4 and L5 DRGs (14 days) from CCI model and sham mice and analyzed their gene expression profiles via RNA‐Seq.

We set a fold change ≥ 2 and a *p* value ≤ 0.05 as the criteria to identify DEGs. On the basis of this standard, we identified 127 DEGs (including 111 upregulated and 16 downregulated DEGs, Additional file 1). Furthermore, a scatter plot (Figure 1E) and a volcano plot (Figure 1F) were used to visualize the DEGs, and a heatmap was used to show the hierarchical clustering of the DEGs (Figure 1G).

To characterize the DEGs presented above, we performed GO enrichment analysis of DEGs in the DRG between the CCI and sham mice. Among the identified DEGs, most genes, including *Npy* [22], *Gal* [23], *Atf3* [24], *Cckbr*, *Adcyap1*, *Vgf*, *Nts*, *Grp*, *Crh*, *Ucn*, and *Prokr2*, have been widely reported to be involved in pain regulation.

By combining our GO analysis results, published DRG tissue gene chip expression data from the selective peripheral nerve injury pain (SNI) mouse model (GEO ID: GSE89224), and recently published transcriptome sequencing data for DRG tissue from rats subjected to oxaliplatin chemotherapy in a peripheral NP model (GSE160543), we identified a novel factor, endothelin‐converting enzyme‐like 1 (*Ecel1*, fold change = 3.55), which is highly likely to be associated with pain.

### 3.2 ECEL1 Expression Is Upregulated in the DRG Following CCI

**FIGURE 2:**
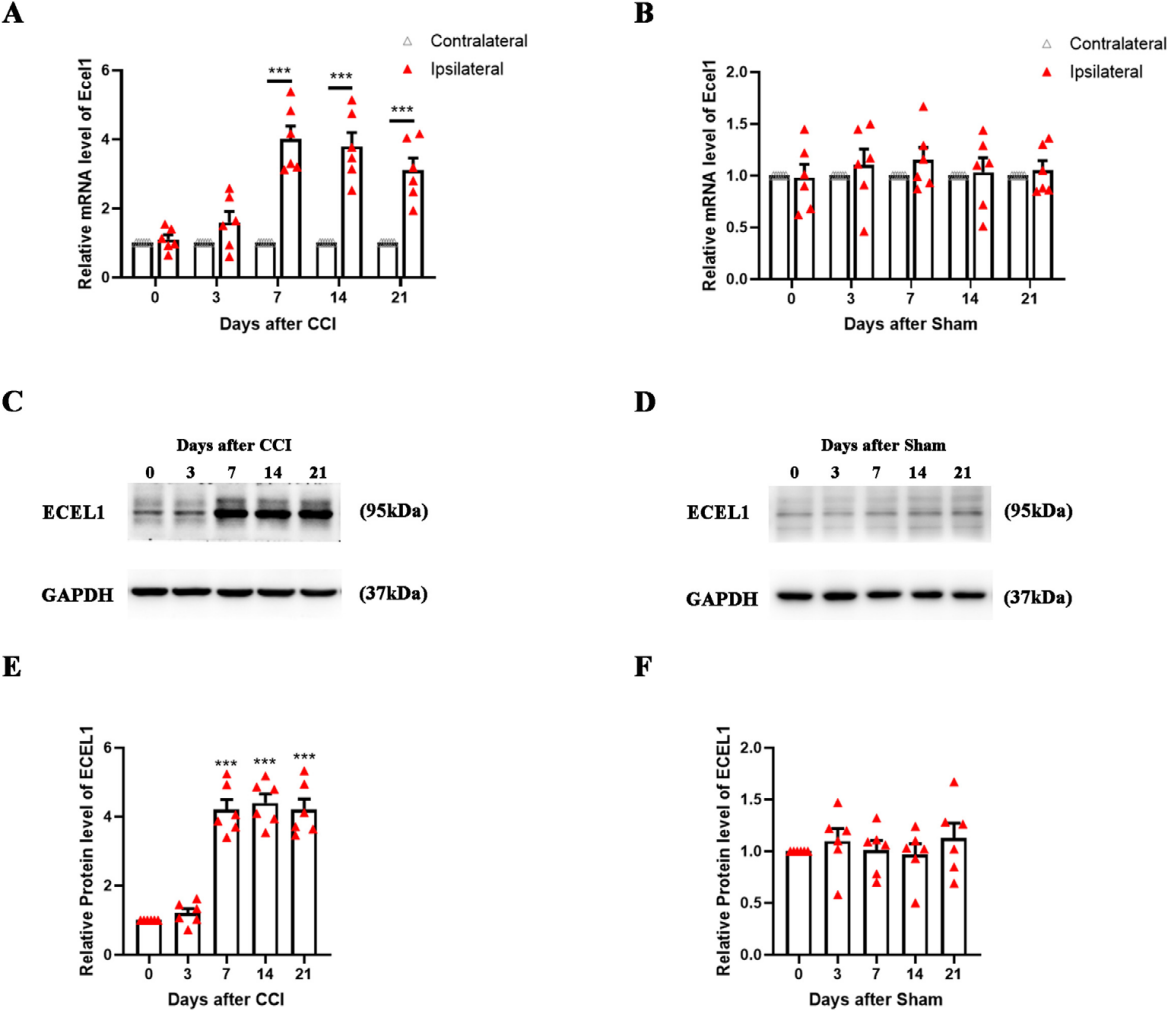
Expression and cellular distributions of ECEL1 in the DRG in mice after CCI. (A, B) Levels of Ecel1 mRNA in the ipsilateral and contralateral L4 and L5 DRGs after sham (A) or CCI (B) surgery. *n* = 6 per group. (C, E) Western blots (C) and statistical analysis (E) of ECEL1 protein levels in the ipsilateral L4 and L5 DRGs after sham surgery. (D, F) Western blots (D) and statistical analysis (F) of ECEL1 protein levels in the ipsilateral L4 and L5 DRGs after CCI. *n* = 6 per group. The results are expressed as the mean ± SEM; *p < 0.05, **p < 0.01, ***p < 0.001 versus the control group. Two‐way ANOVA with Tukey's post hoc test was used in A and B. One‐way ANOVA with Tukey's post hoc test was used in E and F.

Peripheral nerve injury leads to alterations in the expression of various molecules in the DRG, including ECEL1. To further elucidate the temporal pattern of ECEL1 expression, we initially utilized qPCR to measure changes in its mRNA levels at different time points after CCI or sham surgery. Compared with those on the contralateral side, the ipsilateral (injured) L4 and L5 DRGs presented significantly greater levels of Ecel1 mRNA on day 7 (fold change: 4.0 ± 0.9; *p* < 0.0001), day 14 (fold change: 3.8 ± 0.9; *p* < 0.0001), and day 21 (fold change: 3.1 ± 0.8; *p* < 0.0001) after CCI (Figure 2A). The control treatment (sham surgery) did not affect Ecel1 mRNA expression in the ipsilateral DRG (Figure 2B). Additionally, compared with those on day 0, ECEL1 protein levels were elevated on days 7 (fold change: 4.2 ± 0.7; *p* < 0.0001), 14 (fold change: 4.4 ± 0.6; *p* < 0.0001), and 21 (fold change: 4.2 ± 0.8; *p* < 0.0001) (Figure 2C,E). Consistent with the observed changes in mRNA expression, sham surgery did not alter ECEL1 protein levels in the DRG (Figure 2D,F). Collectively, our findings indicate that ECEL1 expression is persistently elevated following CCI.

### 3.3 ECEL1 Knockdown in the DRG Improves CCI‐Induced Hyperalgesia

**FIGURE 3:**
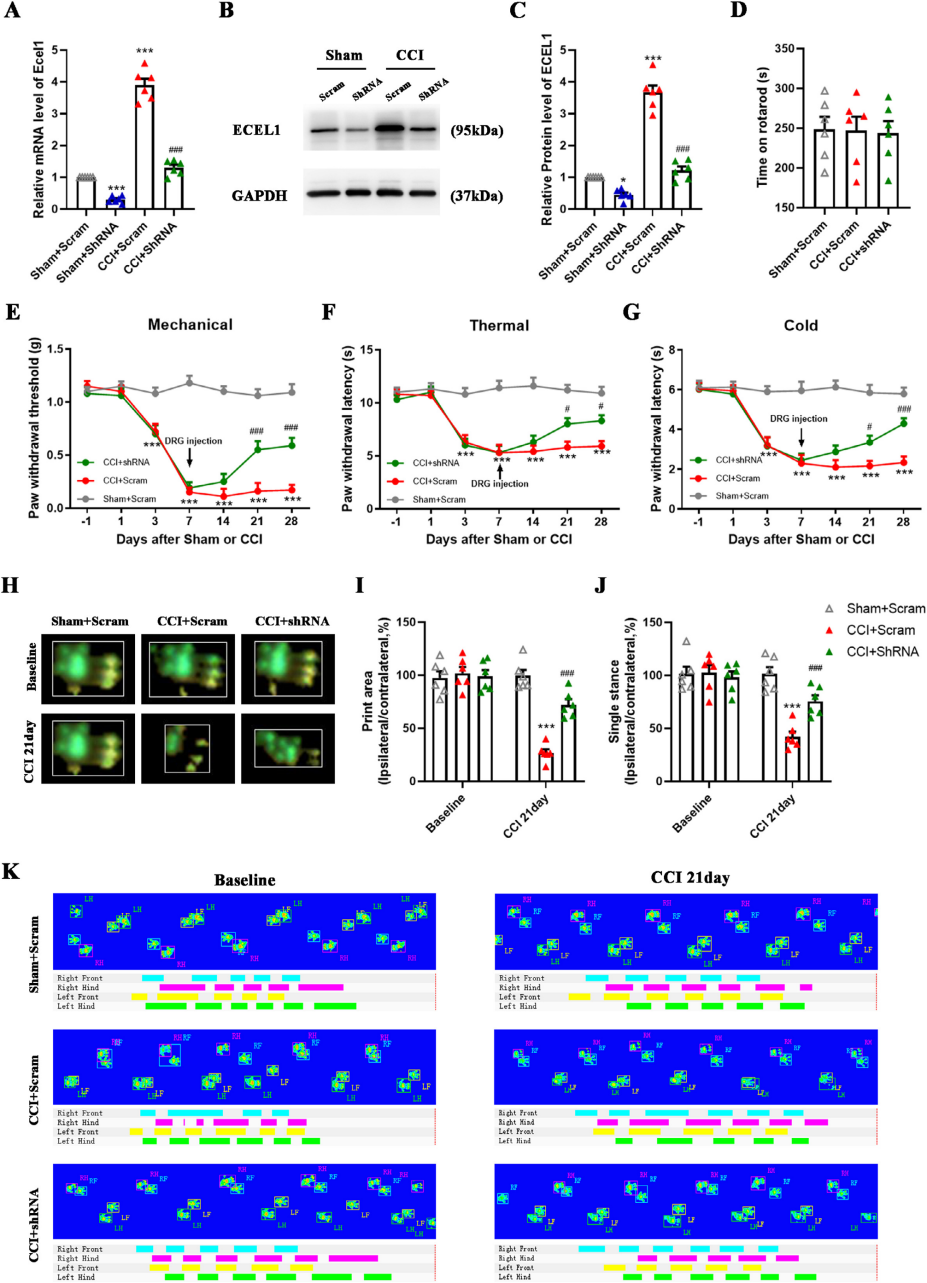
DRG ECEL1 knockdown attenuates CCI injury‐induced nociception development in mice. (A) Levels of Ecel1 mRNA in the L4 and L5 DRGs of mice that were injected with shRNA or scrambled shRNA on day 21 after sham or CCI surgery. (B, C) Western blots (B) and statistical analysis (C) of ECEL1 in the L4 and L5 DRGs of each group of mice. (D) Locomotor performance in the rotarod test of different groups of mice on day 21 after sham or CCI surgery. (E–G) Effects of microinjection of shRNA or Scram into the ipsilateral L4 and L5 DRGs on paw withdrawal responses to mechanical (E), thermal (F), and cold (G) stimuli on the ipsilateral side on the indicated days before or after CCI surgery in mice. (I and J) Percentages of the ipsilateral paw print area (I) and single stance (J) assessed by CatWalk analysis (% ipsilateral/contralateral). (H) Combined paw print image. (K) Representative digitized paw prints and associated step cycles. The results are expressed as the means ± SEMs; *p < 0.05, **p < 0.01, ***p < 0.001 versus the sham + Scram group; ^#^ *p* < 0.05, ^##^ *p* < 0.01, ^###^ *p* < 0.001 versus the CCI + Scram group. One‐way ANOVA with Tukey's post hoc test was used in A, C, and D. Two‐way ANOVA with Tukey's post hoc test was used in E‐G, I, and J. *n* = 6 per group.

To investigate the impact of ECEL1 on nociception following CCI in the DRG and explore the potential therapeutic benefits of ECEL1 knockdown‐mediated alleviation of established nociceptive responses, we administered adeno‐associated virus 5 (AAV5) carrying Ecel1 shRNA into the ipsilateral L4 and L5 DRGs 7 days after CCI surgery. As expected, the Ecel1 shRNA reduced basal ECEL1 expression in the sham group (fold change: 0.45 ± 0.15; *p* = 0.018) and prevented the elevation of ECEL1 protein (CCI + Scram: 3.7 ± 0.5 vs. CCI + shRNA: 1.2 ± 0.3; *p* < 0.0001) and mRNA (CCI + Scram: 3.9 ± 0.4 vs. CCI + shRNA: 1.3 ± 0.2; *p* < 0.0001) expression levels in the DRG on day 21 post‐CCI (Figure 3A–C).

Our findings demonstrated that CCI‐induced mechanical allodynia (Figure 3E), thermal hyperalgesia (Figure 3F), and cold hyperalgesia (Figure 3G) were attenuated 2 weeks after DRG injection of Ecel1 shRNA. However, the rotarod test showed that DRG injection or knockdown of ECEL1 did not alter mouse motor activity (Figure 3D). We also repeated the experiments in mice of different genders, respectively. The results showed that there was no significant difference in the effects of knocking down ECEL1 in the DRG of male and female mice on their neuropathic pain hypersensitivity (Figure S1). Additionally, we utilized CatWalk gait analysis to assess nonreflexive nociceptive behavior following the knockdown of ECEL1 in the DRG. We observed changes in two parameters in the ipsilateral hind paw of mice with CCI: the print area and the single stance (% ipsilateral/contralateral). Representative images show the print area for mice in the sham + Scram, CCI + Scram, and CCI + shRNA groups (Figure 3H,K). The results indicated that DRG injection of Ecel1 shRNA reversed the CCI‐induced reductions in the percentage of print area (CCI + Scram: 26.7% ± 7.6% vs. CCI + shRNA: 72.2% ± 11.3%; *p* < 0.0001; Figure 3I) and single stance (CCI + Scram: 42.2% ± 10.6% vs. CCI + shRNA: 75.6% ± 12.5%; *p* < 0.005; Figure 3J). In summary, our results indicate that the knockdown of ECEL1 can alleviate hyperalgesia induced by CCI.

### 3.4 ECEL1 Knockdown Reduces Neuronal Excitability in the Injured DRG

**FIGURE 4:**
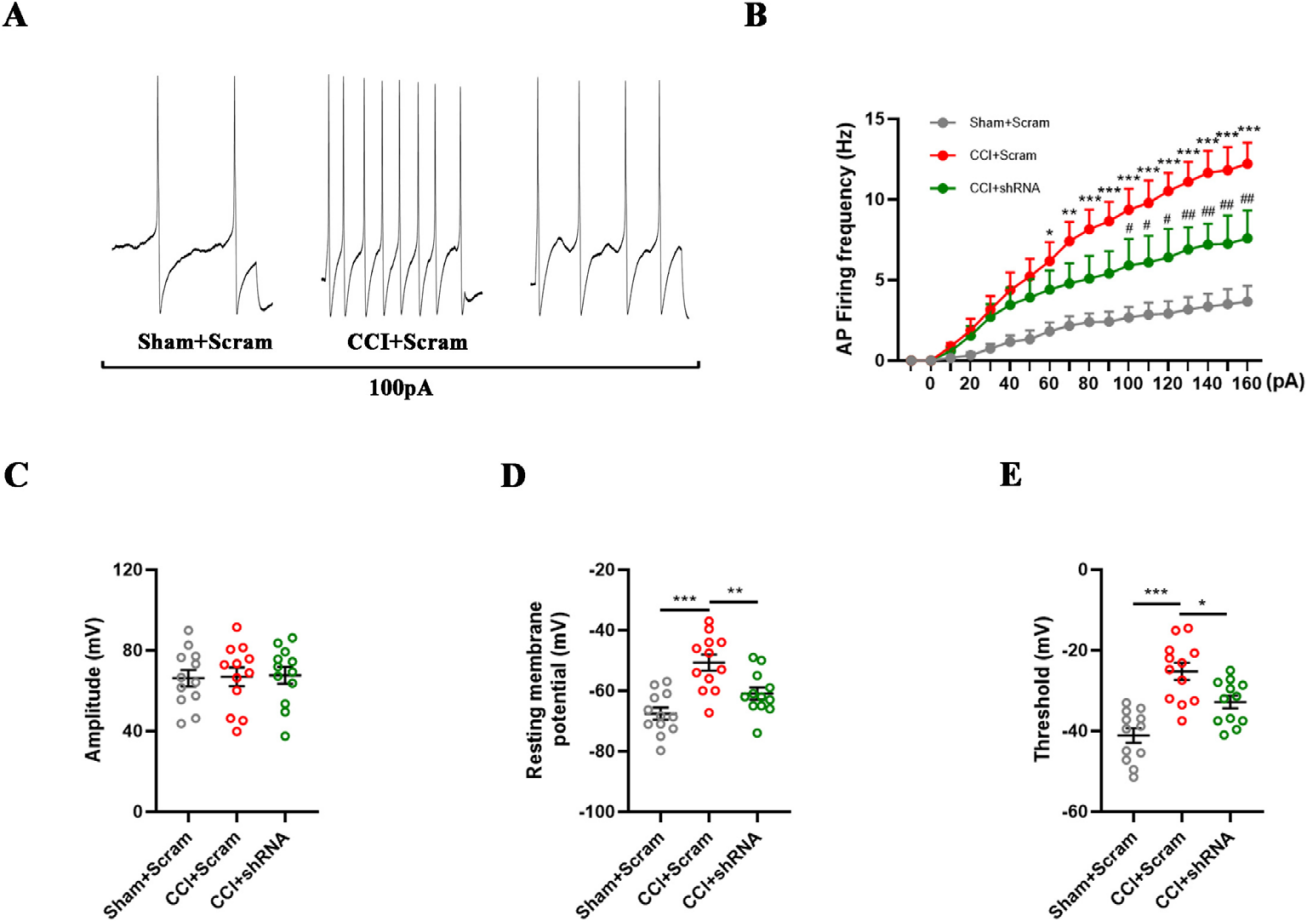
Specific downregulation of ECEL1 alters the excitability of DRG neurons in CCI mice. (A) Current–clamp recordings of the action potential traces of GFP‐positive neurons in DRGs collected from CCI mice 2 weeks after the injection of shRNA or Scram. (B) Quantification of the firing frequency of action potentials in GFP‐positive neurons in the DRG in CCI mice injected with Ecel1 shRNA and Scram. (C–E) Quantification of the amplitude (C), resting membrane potential (D), and threshold of action potential generation (E) in GFP‐positive DRG neurons from different groups of mice. The results are expressed as the means ± SEMs; *p < 0.05, **p < 0.01, ***p < 0.001 versus the Sham + Scram group; #*p* < 0.05, ##*p* < 0.01 versus the CCI + Scram group. Two‐way ANOVA with Tukey's post hoc test was used in B. One‐way ANOVA with Tukey's post hoc test was used in C–E; *n* = 12 per group.

To determine whether the effect of ECEL1 on the nociceptive responses of injured DRG neurons could be attributed to its influence on neuronal excitability, we injected Ecel1 shRNA into the L4 and L5 DRGs of mice on the 7th day after CCI surgery. Two weeks later, whole‐cell current clamp recording was performed on the neurons in the DRG, and the action potentials and resting membrane potentials of the neurons were evaluated. Compared with that in the sham group, the firing rate of action potentials in neurons in the CCI + Scram group was significantly greater, but the injection of Ecel1 shRNA significantly reduced the increasing firing frequency (Figure 4A,B). However, there was no difference in the action potential amplitude between the groups (*p* > 0.05, Figure 4C). In addition, the injection of Ecel1 shRNA resulted in hyperpolarization of the resting membrane potential (CCI + Scram: −50.7 ± 9.2 mV vs. CCI + shRNA: −60.9 ± 6.9 mV; *p* = 0.008; Figure 4D) and an increase in the action potential threshold (CCI + Scram: −25.2 ± 7.4 mV vs. CCI + shRNA: −32.8 ± 5.4 mV; *p* = 0.018; Figure 4E). Overall, these results indicate that ECEL1 can regulate the excitability of injured DRG neurons and that knocking down ECEL1 can reduce the abnormal firing of injured DRG neurons.

### 3.5 Overexpression of ECEL1 in the DRG Leads to Hypersensitivity

**FIGURE 5:**
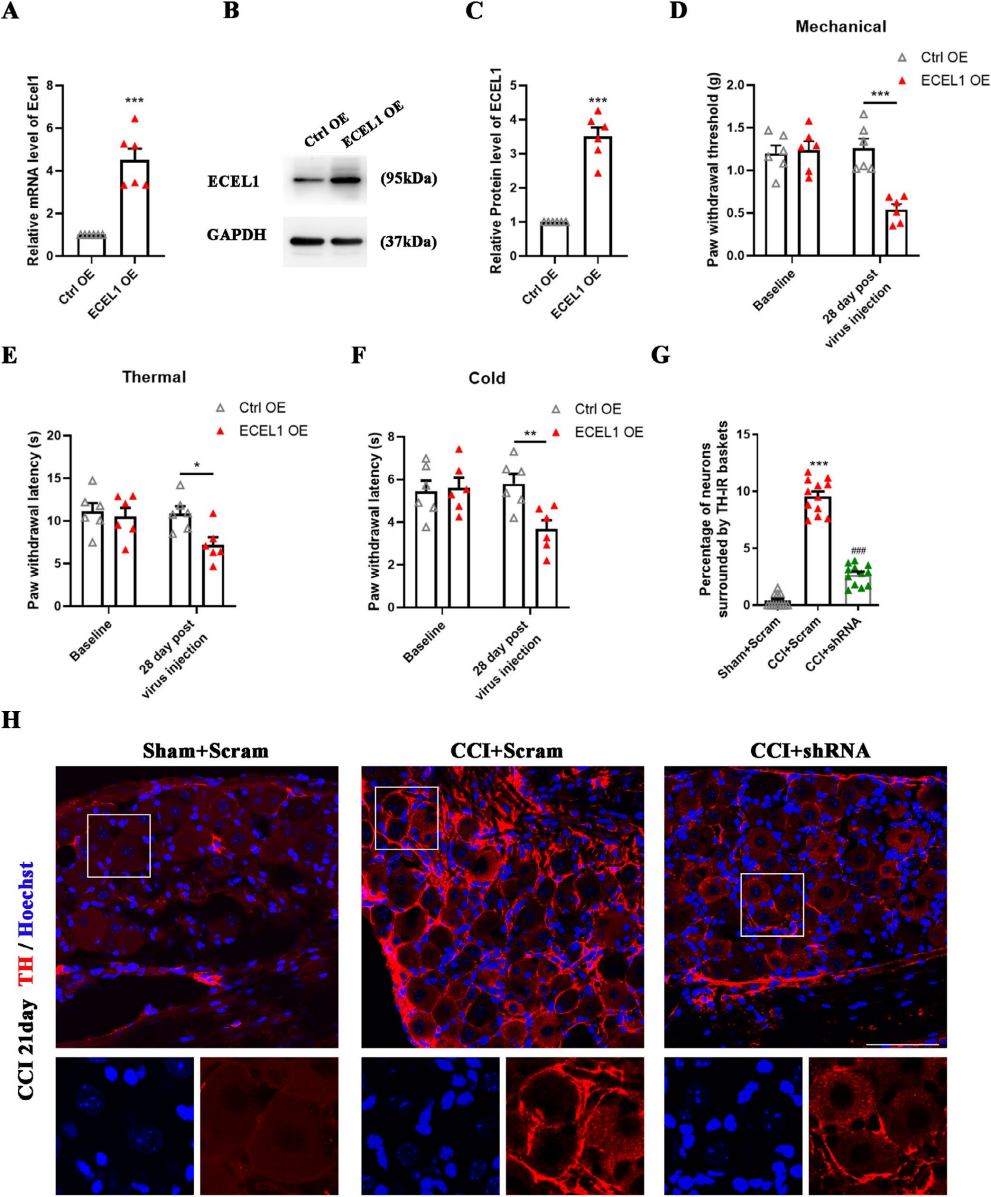
ECEL1 overexpression in sensory neurons of the DRG evokes hyperalgesia symptoms in naive mice. (A–C) Levels of *Ecel1* mRNA (A) and protein (B, C) in the ipsilateral L4 and L5 DRGs after microinjection of an AAV5‐Ecel1‐overexpressing virus or an AAV5‐GFP control virus into the unilateral L4 and L5 DRGs in mice. *N* = 6 per group. (D–F) Paw withdrawal responses to mechanical (D), thermal €, and cold (F) stimuli on the ipsilateral side after microinjection of an ECEL1‐overexpressing strain and a GFP virus into the ipsilateral L4 and L5 DRGs in naive mice. *n* = 6 per group. (H) Representative images showing the basket structures (red) around DRG neurons in each group of mice (scale bar, 50 μm). (G) Statistical analysis of the percentage of DRG neurons surrounded by TH‐IR baskets. *n* = 12 per group. The results are expressed as the mean ± SEM; *p < 0.05, **p < 0.01, ***p < 0.001 versus the sham or control group, ^###^ *p* < 0.001 versus the CCI + Scram group. Student's t test was used in A, C. Two‐way ANOVA with Tukey's post hoc test was used in D–F. One‐way ANOVA with Tukey's post hoc test was used in G.

To further validate the ability of elevated ECEL1 levels in the DRG following early‐stage nerve injury to induce nociception, we administered AAV5‐Ecel1, which expresses full‐length Ecel1, into the L4 and L5 DRGs of naive mice. AAV5‐Ctrl was utilized as a control. Four weeks post‐injection, both Ecel1 mRNA (fold change: 4.5 ± 1.2; *p* < 0.0001; Figure 5A) and ECEL1 protein (fold change: 3.5 ± 0.6; *p* < 0.0001; Figure 5B,C) expression levels in the DRG in AAV5‐Ecel1‐injected mice were greater than those in the DRG in control mice. Compared with those injected with AAV5‐Ctrl, mice injected with AAV5‐Ecel1 presented lower paw withdrawal thresholds during mechanical stimulation (Ctrl OE: 1.26 ± 0.25 g vs. ECEL1 OE: 0.54 ± 0.14 g; *p* < 0.0001; Figure 5D) and reduced paw withdrawal latencies during thermal (Ctrl OE: 10.88 ± 1.87 s vs. ECEL1 OE: 7.21 ± 1.92 s; *p* = 0.0127; Figure 5E) and cold (Ctrl OE: 5.8 ± 1.03 s vs. ECEL1 OE: 3.67 ± 0.94 s; *p* = 0.0039; Figure 5F) stimulation. These findings suggest that the overexpression of ECEL1 in the DRG in naive mice leads to symptoms resembling those associated with nociception, including mechanical allodynia and heightened responses to thermal and cold stimuli.

### 3.6 ECEL1 Promotes Sympathetic Sprouting, Which Leads to NP, and Is Regulated by LIF

**FIGURE 6:**
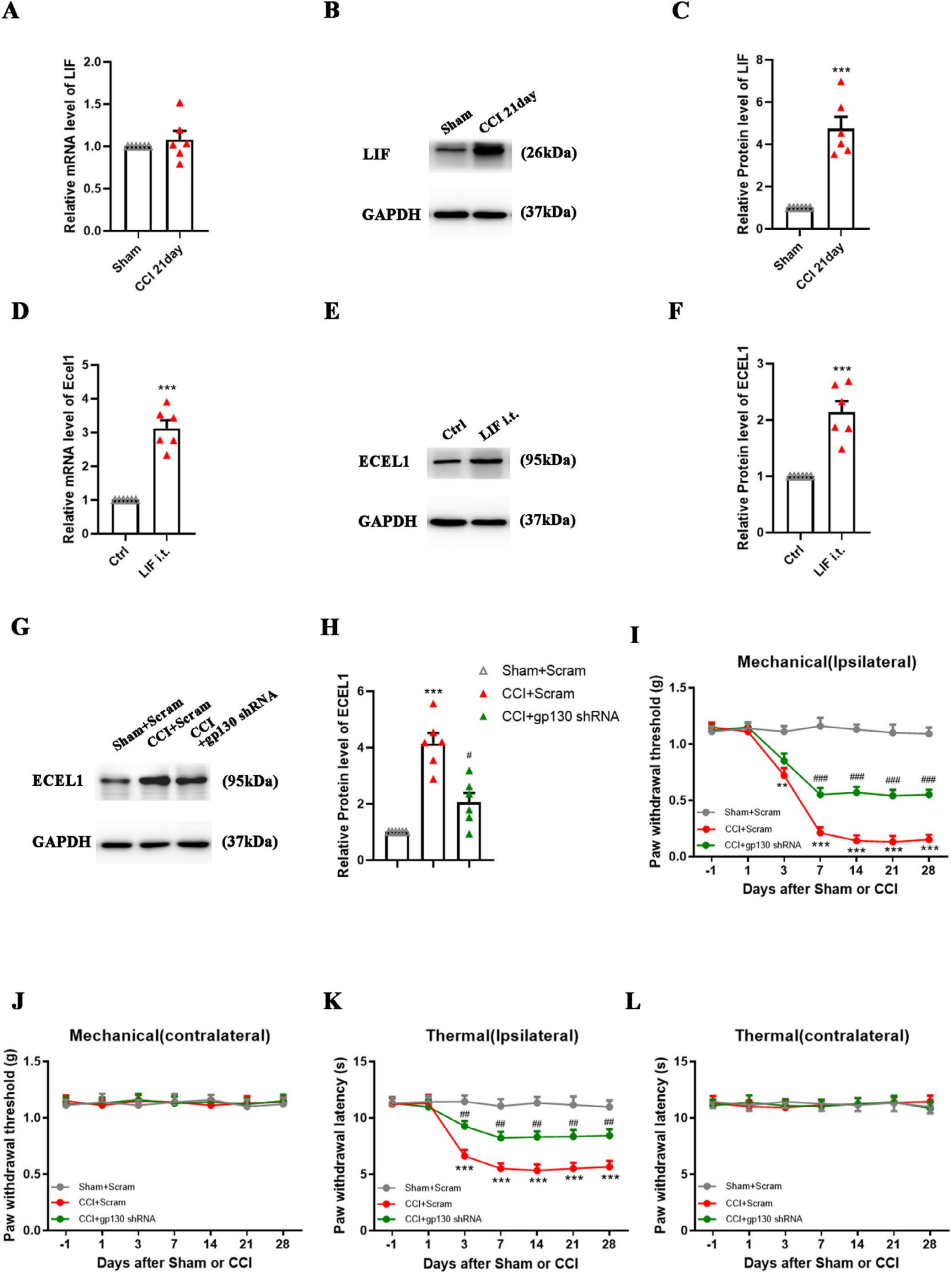
ECEL1 is regulated by LIF. (A, B) Western blots (A) and statistical analysis (B) of LIF protein levels in the DRG after CCI. *n* = 6 per group. (C, D) Western blots (C) and statistical analysis (D) of the expression of ECEL1 in the DRG in mice injected with gp130 shRNA or Scram 14 days before sham or CCI surgery. (E–G) ECEL1 protein levels (E, F) and quantitative PCR assays of ECEL1 mRNA levels (G) in the DRG after the intrathecal injection of LIF. *n* = 6 per group. (H–K) Paw withdrawal responses to mechanical (H, I) and thermal (J, K) stimuli on the ipsilateral side and contralateral side in CCI or sham mice injected with gp130 shRNA or Scram. *n* = 6 per group. The results are expressed as the mean ± SEM; ***p < 0.005 versus the sham or control group; ^##^ *p* < 0.01 and ^###^ *p* < 0.001 versus the CCI + Scram group. Student's t test was used in B, F, G. One‐way ANOVA with Tukey's post hoc test was used in D. Two‐way ANOVA with Tukey's post hoc test was used in H–K.

In the aforementioned results, it has been established that knockdown of ECEL1 in CCI mice effectively alleviates symptoms associated with NP. However, the mechanism by which ECEL1 exerts its regulatory function in the context of NP remains to be elucidated. To gain insights into the underlying mechanisms, we directed our attention to the formation of sympathetic basket structures (SBF) surrounding DRG neurons. Therefore, in this study, we used TH‐IR staining to detect basket structures around DRG neurons in each group of mice (Figure 5H). Compared with that in the sham group, the proportion of DRG neurons with basket formation among all neurons was significantly greater in the CCI group, and Ecel1 knockdown reduced this proportion (CCI + Scram: 9.6% ± 1.5% vs. CCI + shRNA: 2.7% ± 0.9%; *p* < 0.001; Figure 5G). These findings suggest that the analgesic effect of Ecel1 knockdown may be achieved by reducing the number of SBFs around DRG neurons. Therefore, we sought to identify the upstream molecule of ECEL1. Studies have shown that the expression of leukemia inhibitory factor (LIF) in the DRG increases after nerve injury [25] and in a rat foot incision surgery model [26], and that adding LIF to rat DRG cells cultured in vitro or intrathecally injecting LIF can promote the expression of Ecel1 [9]. To verify this result, we detected the expression of LIF in the DRG on day 21 post‐CCI and observed that although its mRNA level did not change significantly (Figure 6A), its protein expression level was markedly increased (fold change: 4.8 ± 1.2; *p* < 0.0001; Figure 6B,C). It suggests that LIF was not produced in the DRG but may be transported from a distal site, such as the site of injury, through axoplasmic transport or blood circulation, etc. We then continuously injected LIF intrathecally into wild‐type mice for 3 days and found that Ecel1 mRNA (fold change: 3.1 ± 0.5; *p* < 0.0001; Figure 6D) and protein expression levels in the DRG increased (fold change: 2.1 ± 0.4; *p* < 0.0001; Figure 6E,F). LIF binds to a receptor that consists of LIF receptor β and gp130 to exert its effects [25]. To block the effect of LIF, we injected gp130 shRNA or control scrambled shRNA (Scram) into the L4 and L5 DRGs of wild‐type mice and then performed CCI surgery 14 days later. Compared with that in the CCI + Scram group, the expression of Ecel1 in the CCI + gp130 shRNA group was significantly lower (fold change: CCI + Scram: 4.2 ± 0.8 vs. CCI + gp130 shRNA: 2.1 ± 0.7; *p* < 0.005; Figure 6G,H), indicating that LIF is an upstream molecule of Ecel1. We also detected nociceptive responses on the ipsilateral (injured) and contralateral sides. Compared with those in the CCI + Scram group, the mechanical allodynia and thermal hyperalgesia of the CCI + gp130 shRNA group were significantly weaker from day 7 post‐CCI, but there was no significant difference between these groups on the contralateral side (Figure 6I–L).

**FIGURE 7:**
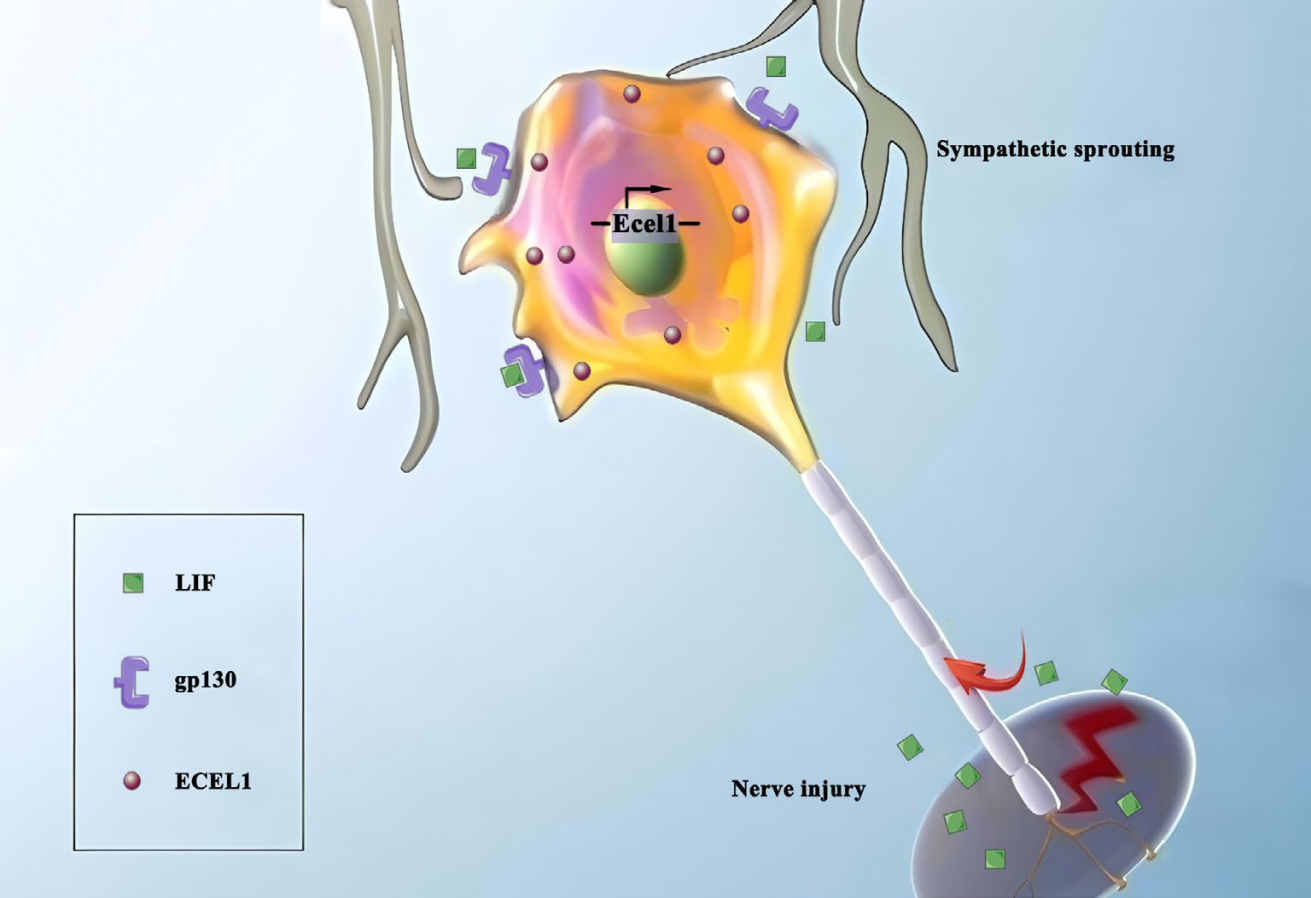
Proposed model of the relationship between peripheral nerve injury‐induced nociception and alterations in ECEL1 expression, as well as the mechanism by which ECEL1 is regulated by LIF.

Thus, based on our findings, we hypothesize that LIF may be transported from the distal injury site to DRG neurons, where it activates downstream signaling to promote ECEL1 expression. Consistent with this model, blocking LIF signaling or knocking down Ecel1 reduces SBF and alleviates CCI‐induced nociception (Figure 7).

## 4 Discussion

ECEL1 is a metallopeptidase that is specific to neurons. ECEL1 is primarily localized in the endoplasmic reticulum. As a membrane‐bound metallopeptidase, ECEL1 has a transmembrane domain, and part of the protein is located on the cell membrane to exert its function [27]. Although its underlying mechanism and substrate are still unknown, ECEL1 structurally resembles other members of the family, such as endothelin‐converting enzyme 1 (ECE1), endothelin‐converting enzyme 2 (ECE2), and neprilysin (NEP), which are all major zinc proteases in the nervous system. They can degrade amyloid‐β peptide (Aβ) in the brain, thus triggering pathologies related to neuronal cell death. This has received much attention recently [28]. Both of these enzymes exhibit indirect neuron‐rescue activity, suggesting that ECEL1 might exert similar effects. Therefore, members of this family, including ECEL1, may play a neuroprotective role [29]. Previous studies have shown that in ischemic brain injury, ECEL1 is expressed in the neurons of the peri‐infarct cortex and thalamus, protecting them from secondary degenerative death [8]. Meanwhile, knocking down Ecel1 in retinal ganglion cells (RGCs) after optic nerve injury accelerates the death of RGCs [13]. In normal rat brain tissue, ECEL1 is concentrated in the hypothalamus, where various neuropeptides are expressed, indicating a strong connection between ECEL1 and neuropeptides. The expression of neuropeptides—some of which are known for their neuroprotective effects—is significantly altered after nerve injury. Our study demonstrated that following CCI, ECEL1 expression is also altered within a specific group of DRG cells. While few uninjured DRG neurons expressed ECEL1 mRNA before injury, after sciatic nerve damage occurred, the mRNA expression of ECEL1 was markedly increased.

Our experiment explored the consequences of negatively regulating ECEL1 in CCI mice. Meanwhile, it also clarified that increasing the expression of ECEL1 under physiological conditions will lead to hyperalgesia. This strongly implies that ECEL1, through its specific functions within the DRG neurons, has the capacity to disrupt the normal physiological processes associated with pain perception. Just as negative regulation of ECEL1 likely affects pain‐related pathways in one way, its overexpression in the physiological state has the opposite effect, further emphasizing the importance of ECEL1 in maintaining the balance of pain perception. This overexpression experiment provides a complementary perspective.

Regarding the mechanism of ECEL's action, first, LIF, the most versatile member of the interleukin‐6 cytokine family, operates through a receptor composed of LIF receptor β and gp130. Depending on the cell type, LIF can have opposing effects, such as promoting or inhibiting proliferation, differentiation, and survival [25]. Studies have shown that LIF can participate in the regeneration of nerves through the transcription factor STAT3 [30], and STAT3 can regulate the expression of ECEL1 [31]. Therefore, we speculate that LIF activates the STAT3 signaling pathway through the gp130 receptor, promoting the expression of ECEL1 and thereby participating in NP. The detailed mechanism remains to be further studied. Another notable point is that in our study, we verified that the expression of Ecel1 decreased significantly after CCI surgery when gp130 shRNA was injected into the DRG in advance. The complex receptor formed by gp130 can bind to other closely related interleukin 6 (IL‐6) family members, such as IL‐6, IL‐11, and ciliary neurotrophic growth factor (CNTF) [32]. While IL‐6 or CNTF might replicate the impact of LIF on peptide induction in sympathetic neurons under certain conditions, there is ample evidence indicating that many biological effects of IL‐6 family cytokines that utilize gp130 as their receptor are not completely interchangeable [33]. To some extent, it is worth considering that ECEL1 might also be regulated by other members of the IL‐6 family. We will continue to explore these questions in our future studies.

Second, in the context of the pathogenesis of neuropathic pain, clinical case studies and animal models have shown a strong connection between sympathetic nervous system disorders and NP. Symptoms like spontaneous pain, hyperalgesia, and trigger pain often accompany sympathetic nervous system disruptions after peripheral nerve injury, and blocking or removing sympathetic nerve fibers can significantly relieve these pain types [34]. McLachlan et al. [35] first reported the abnormal growth of sympathetic nerve fibers in the DRG after peripheral nerve injury, called “sympathetic sprouting,” which results in the formation of SBF around sensory neurons. The abnormal contact between sympathetic and sensory neurons is thought to trigger abnormal discharges after peripheral nerve injury, which may be an essential factor in the development and maintenance of NP [36]. In our study, we observed an increase in SBF in the CCI group, and knocking down Ecel1 reduced this proportion. It is possible that the upregulated ECEL1, regulated by LIF, is linked to the processes of sympathetic sprouting and SBF formation. ECEL1 may modulate the molecular and cellular events that promote sympathetic–sensory neuron interactions, for example, by affecting the expression or activity of molecules involved in axonal guidance or synaptic plasticity, leading to enhanced SBF formation.

In conclusion, our study indicates that ECEL1 in the injured DRG induces hyperalgesia by promoting the formation of sympathetic basket structures after peripheral nerve injury. We demonstrated that blocking the increased expression of ECEL1 in the DRG caused by peripheral nerve injury could decrease the high excitability of injured DRG neurons and attenuate nociceptive sensation. However, one limitation is that our findings were obtained in a mouse model rather than in patients with chronic pain. Although experiments conducted on mice offer the benefit of obtaining a detailed mechanistic understanding, the applicability of these results to human pain responses must be verified in the future. Nevertheless, the novel knowledge obtained in this study enhances our understanding of the transcriptional mechanisms underlying the emergence of nociceptive perception induced by peripheral nerve injury, suggesting that ECEL1 might be a new target for the prevention and treatment of NP.

## Conflicts of Interest

The authors declare no conflicts of interest.

## Supporting information


**Figure S1:** The impact of DRG ECEL1 knockdown in mice of different genders on their nociceptive response thresholds. (A) Locomotor performance in the rotarod test of different genders of mice on day 21 after CCI surgery. (B‐D) Effects of microinjection of shRNA or Scram into the ipsilateral L4 and L5 DRGs on paw withdrawal responses to mechanical (B), thermal (C), and cold (D) stimuli on the ipsilateral side on the indicated days before or after CCI surgery in male and female mice. There was no statistical difference between CCI + shRNA (male) group and CCI + shRNA (female) group. The results are expressed as the means ± SEMs; One‐way ANOVA with Tukey's post hoc test was used in A. Two‐way ANOVA with Tukey's post hoc test was used in B–D. *n* = 6 per group.

**Figure S2:** The original blot images are involved in the article.

**Additional file 1:** cns70578‐sup‐0003‐Additional‐file‐1

## Data Availability

The data that support the findings of this study are available on request from the corresponding author. The data are not publicly available due to privacy or ethical restrictions.
