# Supplementary figures and images for: Endothelin‐Converting Enzyme‐Like 1 Regulated by LIF Contributes to Chronic Constriction Injury‐Induced Neuropathic Pain in Mice
Source: CNS Neurosci Ther. 2025 Sep 4;31(9):e70578. doi: 10.1111/cns.70578 (PMC12409069; doi:10.1111/cns.70578)

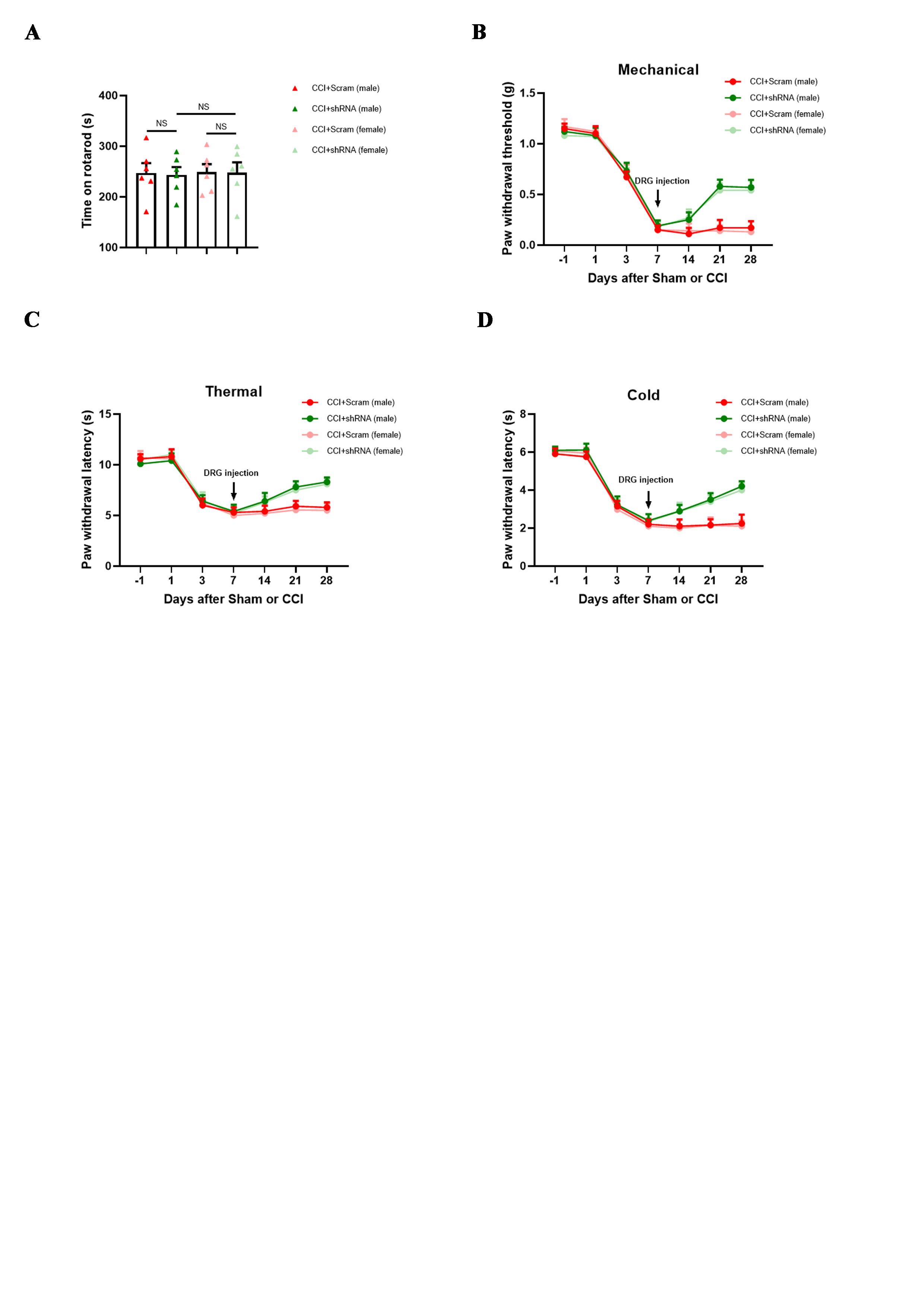

Supplement: Supplementary file 1 — Figure S1: The impact of DRG ECEL1 knockdown in mice of different genders on their nociceptive response thresholds. (A) Locomotor performance in the rotarod test of different genders of mice on day 21 after CCI surgery. (B‐D) Effects of microinjection of shRNA or Scram into the ipsilateral L4 and L5 DRGs on paw withdrawal responses to mechanical (B), thermal (C), and cold (D) stimuli on the ipsilateral side on the indicated days before or after CCI surgery in male and female mice. There was no statistical difference between CCI + shRNA (male) group and CCI + shRNA (female) group. The results are expressed as the means ± SEMs; One‐way ANOVA with Tukey's post hoc test was used in A. Two‐way ANOVA with Tukey's post hoc test was used in B–D. n = 6 per group. [file CNS-31-e70578-s003.tif]

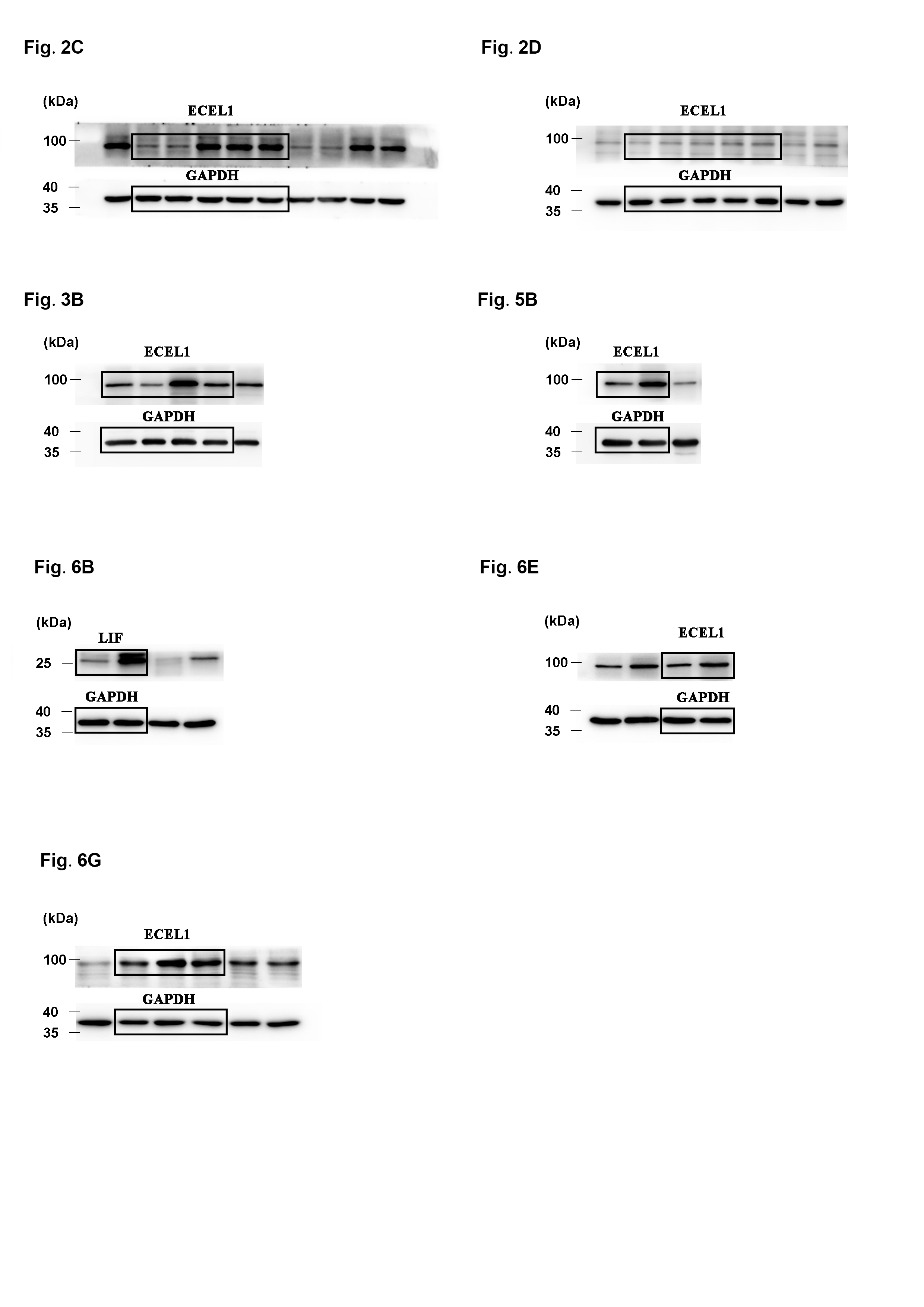

Supplement: Supplementary file 2 — Figure S2: The original blot images are involved in the article. [file CNS-31-e70578-s001.tif]
